# Antibacterial and Immunomodulatory Effects of *Withania somnifera* Ethanolic Root Extract Against Colistin-Resistant *Acinetobacter baumannii* in Leukopenic Mice

**DOI:** 10.3390/ijms27167321

**Published:** 2026-08-16

**Authors:** Masood Alam Khan, Hafiz Iqtidar Ahmad, Mohd Azam, Mohd Masih Uzzaman Khan, Ahmad N. Aljarbou, Arif Khan

**Affiliations:** 1 Department of Basic Health Sciences, College of Applied Medical Sciences, Qassim University, Buraidah 51452, Saudi Arabia; 4140@qu.edu.sa; 2 Department of Tashreeh Wa Munafeul Aza, Faculty of Unani Medicine, Aligarh Muslim University, Aligarh 202002, India; hafiziqtidar@gmail.com; 3 Department of Medical Laboratories, College of Applied Medical Sciences, Qassim University, Buraidah 51452, Saudi Arabia; m.aftab@qu.edu.sa; 4 Department of Pharmaceutical Chemistry & Pharmacognosy, College of Pharmacy, Qassim University, Buraydah 51452, Saudi Arabia; mo.khan@qu.edu.sa; 5 Department of Pharmaceutics, College of Pharmacy, Qassim University, Buraydah 51452, Saudi Arabia; ajrboa@qu.edu.sa

**Keywords:** drug resistance, immunity, infection, Ashwagandha, antimicrobial activity

## Abstract

*Acinetobacter baumannii*, particularly colistin-resistant strains, represents a major therapeutic challenge because of multidrug resistance, biofilm formation, and limited treatment options. This study investigated the antibacterial, antibiofilm, immunomodulatory, and therapeutic potential of *Withania somnifera* ethanolic root extracts (WSEEs) against drug-sensitive (DS) and colistin-resistant (CR) *A. baumannii*. WSEE exhibited concentration-dependent antibacterial activity, with MIC/MBC values of 125/250 μg/mL against the DS isolate and 250/500 μg/mL against the CR isolate. Time–kill analysis demonstrated bactericidal activity at 250 μg/mL, and WSEE significantly reduced the biomass of preformed biofilms. In cyclophosphamide-induced leukopenic mice, oral administration of WSEE (100 and 200 mg/kg) restored leukocyte counts and significantly reduced pulmonary bacterial burden following infection with both isolates. The higher dose (200 mg/kg) achieved survival rates of 90% and 70% in mice infected with the DS and CR isolates, respectively, compared with 50% and 30% in the corresponding colistin-treated groups. WSEE also significantly decreased serum levels of the pro-inflammatory cytokines, such as tumor necrosis factor-alpha (TNF-α) and interleukin-6 (IL-6), indicating attenuation of infection-associated inflammation. Biochemical analyses further showed that WSEE did not produce detectable hepatotoxicity or nephrotoxicity under the experimental conditions. Collectively, these findings demonstrate that WSEE possesses antibacterial, antibiofilm, anti-inflammatory, and immunomodulatory activities and provides therapeutic benefit in experimental *A. baumannii* infection. These results support the potential of WSEE as a complementary therapeutic strategy against multidrug-resistant *A. baumannii*, while further mechanistic, pharmacokinetic, and translational studies are needed to validate its clinical potential.

## 1. Introduction

*Acinetobacter baumannii* is a Gram-negative opportunistic pathogen that has become a major global health concern due to its high adaptability and environmental persistence [1,2]. Once considered low virulence, it is now a leading cause of hospital-acquired infections such as pneumonia, septicemia, and wound infections, especially in critically ill and immunocompromised patients [3]. Its rapid and sustained acquisition of multidrug resistance, notably to carbapenems, has severely limited available treatment options and complicated clinical management [4,5]. In addition, its ability to form biofilms on medical devices and hospital surfaces enhances persistence, facilitates hospital transmission, and contributes to treatment failure [6,7]. Collectively, these features underscore the clinical importance of *A. baumannii* and highlight the urgent need to better understand its biology, virulence determinants, and antimicrobial resistance mechanisms. These insights are vital for guiding effective infection control practices and advancing therapeutic strategies against this pathogen.

The genome of *A. baumannii* encodes multiple antimicrobial resistance molecules, including β-lactamases, aminoglycoside-modifying enzymes, and multidrug efflux pumps, which together drive its strong drug-resistant phenotype [8,9]. Of particular concern are carbapenemase-producing strains expressing OXA-type β-lactamases (e.g., OXA-23, OXA-24, OXA-58), which severely limit treatment options and are linked to poor clinical outcomes [10,11]. A clear understanding of these resistance mechanisms is essential for developing targeted therapies to overcome antimicrobial resistance. Given the rising global burden of multidrug-resistant *A. baumannii*, increasing attention is being directed toward alternative and complementary approaches, including herbal and plant-derived therapies. Several plant extracts have shown notable antibacterial activity against *A. baumannii*, supporting their potential use as adjuncts or alternatives to conventional antibiotics [12,13,14]. Importantly, phytochemicals can also act synergistically with existing antibiotics, enhancing their efficacy and, in some cases, restoring activity against resistant strains. Compounds such as thymoquinone, ellagic acid, and berberine have shown promising therapeutic effects in experimental models of *A. baumannii* infection, underscoring the translational potential of plant-based bioactives in combating MDR pathogens [15,16,17].

Among traditional medicinal plants, *W. somnifera* (L.) Dunal (Ashwagandha) has gained significant scientific attention due to its wide range of pharmacological activities, including antioxidant, anti-inflammatory, antimicrobial, anticancer, neuroprotective, and immunomodulatory effects [18,19,20,21]. In Ayurvedic medicine, *W. somnifera* (Ashwagandha) is recognized as an adaptogenic herb with potent anti-inflammatory and immunomodulatory activities. Experimental and clinical studies have shown that Ashwagandha alleviates physiological stress by lowering serum cortisol levels [22], improves physical performance and vitality [23], reduces pro-inflammatory cytokines and oxidative stress [24], enhances sleep quality [25], promotes immune function through increased leukocyte proliferation and natural killer (NK) cell activity [26], and supports reproductive health [27]. In the context of infectious diseases, withanolides derived from *W. somnifera* exhibit antiviral activity and can augment host immune responses [28]. Its historical use in the management of chronic infections, including tuberculosis, underscores its potent immunostimulatory and adaptogenic properties [29]. Moreover, *W. somnifera* root extract has demonstrated antifungal activity against *Candida albicans*, supporting its broad-spectrum antimicrobial potential [30].

Although *W. somnifera* possesses well-documented antimicrobial and immunomodulatory properties, its therapeutic efficacy against CR *A. baumannii* infection in immunocompromised hosts has not been systematically investigated. In particular, its ability to simultaneously inhibit CR *A. baumannii*, disrupt biofilm formation, and improve infection outcomes under immunosuppressed conditions remains insufficiently defined. Therefore, the present study was designed to evaluate the antibacterial, antibiofilm, immunomodulatory, and therapeutic efficacy of *W. somnifera* ethanolic extract (WSEE) against both DS and CR *A. baumannii* using in vitro assays and a cyclophosphamide-induced leukopenic mouse model. WSEE exhibited significant antibacterial and antibiofilm activities in vitro and provided marked protection in vivo by reducing bacterial burden and improving survival in leukopenic mice challenged with both bacterial strains. These protective effects were accompanied by enhanced leukocyte recovery and reduced serum levels of the pro-inflammatory cytokines such as TNF-α and IL-6, indicating immunomodulatory activity. Collectively, these findings suggest that WSEE may represent a promising complementary therapeutic strategy for the management of drug-resistant *A. baumannii* infections.

## 2. Results

### 2.1. GC–MS Characterization of WSEE

Gas chromatography–mass spectrometry (GC–MS) profiling of WSEE demonstrated the presence of a broad and complex array of secondary metabolites with recognized biological significance (Supplementary Figure S1). Many of the detected constituents possess reported antimicrobial, immunomodulatory, anti-inflammatory, and antioxidant properties (Table 1). The identification of these bioactive compounds substantiates the traditional therapeutic applications of *W. somnifera* and underscores its value as a source of multifunctional phytochemicals. Collectively, the GC–MS-based chemical characterization provides a preliminary phytochemical basis that may contribute to the antibacterial and immunomodulatory activities of WSEE observed in subsequent studies. The GC–MS analysis was performed to provide qualitative phytochemical characterization of the extract, while identification of the specific bioactive compounds responsible for the observed biological activities warrants further investigation using HPLC–MS-guided analyses.

### 2.2. Antioxidant Capacity and Total Phenolic and Flavonoid Contents of WSEE

The antioxidant potential of the WSEE was evaluated using a DPPH free radical scavenging assay. The WSEE demonstrated appreciable free radical scavenging activity, with an IC_50_ value of 128.8 ± 6.2 μg/mL. To further characterize its phytochemical composition, the TPC and TFC were determined using the Folin–Ciocalteu and aluminum chloride colorimetric methods, respectively. The TPC, expressed as milligrams of gallic acid equivalents (GAE) per gram of extract, was 95.5 ± 7.4 mg GAE/g, whereas the TFC, expressed as milligrams of quercetin equivalents (QE) per gram of extract, was 21.2 mg QE/g. These findings indicate that WSEE is a rich source of phenolic and flavonoid compounds, which may contribute to its antimicrobial properties (Table 1).

### 2.3. WSEE Demonstrated Potent Activity Against CR A. baumannii

**Figure 1:**
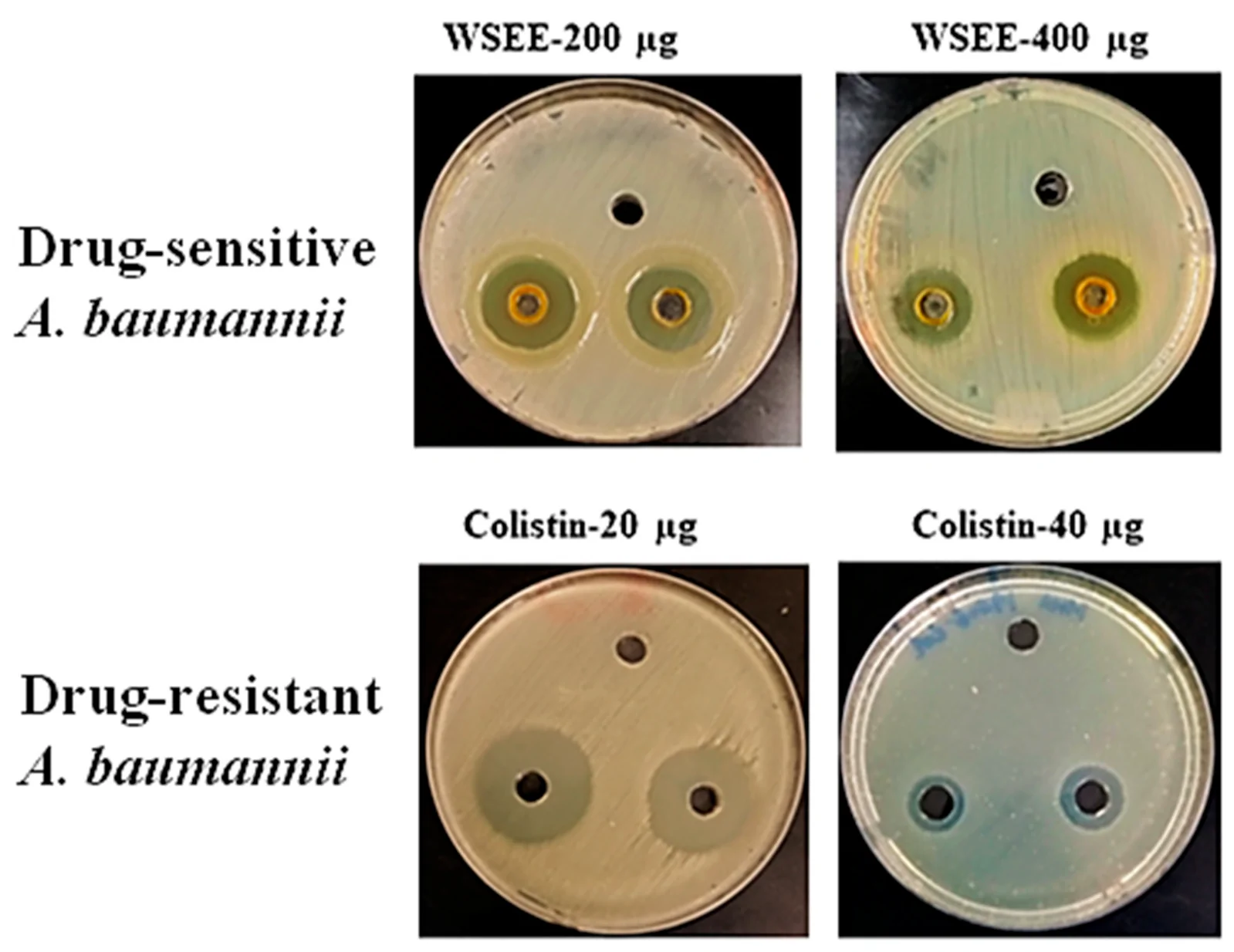
**Antibacterial efficacy of WSEE against DS and CR *A. baumannii*.** Representative agar well diffusion assay showing zones of inhibition produced by WSEE at 200 µg and 400 µg against DS *A. baumannii* (upper panels). The lower panels depict the activity of colistin (20 µg and 40 µg) against the CR *A. baumannii* isolate. WSEE exhibits clear, concentration-dependent inhibition against both DS and CR strains, whereas colistin shows markedly reduced activity against the CR isolate.

WSEE demonstrated strong activity against both DS and CR *A. baumannii* isolates. As shown in Figure 1, WSEE suppressed bacterial growth regardless of resistance status, whereas colistin was largely effective only against the DS strain. Treatment with WSEE at 200 µg and 400 µg resulted in inhibition zones of 17 mm and 26 mm, respectively, against the DS isolate. Importantly, similar inhibitory effects were observed against the CR isolate, with zones of 14 mm and 23 mm at the corresponding concentrations.

In contrast, colistin exhibited greater potency against the DS isolate, producing inhibition zones of 27 mm at 20 µg and 32 mm at 40 µg. However, its activity was substantially compromised against the resistant strain, where inhibition zones were limited to 9 mm and 12 mm at the same doses. The MIC values for WSEE against the DS and CR *A. baumannii* strains were 125 µg/mL and 250 µg/mL, respectively. The corresponding MBC values were 250 µg/mL and 500 µg/mL, indicating bactericidal activity at concentrations two-fold higher than the MICs. For colistin, the MIC was 1 µg/mL against the DS strain and >4 µg/mL against the CS isolate. The MBC of colistin was 1 µg/mL against the DS strain, while the MBC exceeded 16 µg/mL for the CR isolate, confirming its markedly reduced susceptibility. Taken together, these findings highlight the broad-spectrum antibacterial activity of WSEE, particularly its effectiveness against CR *A. baumannii*, supporting its potential as a complementary or alternative strategy for managing multidrug-resistant infections.

### 2.4. WSEE Demonstrated Bactericidal Action Against CR A. baumannii

**Figure 2:**
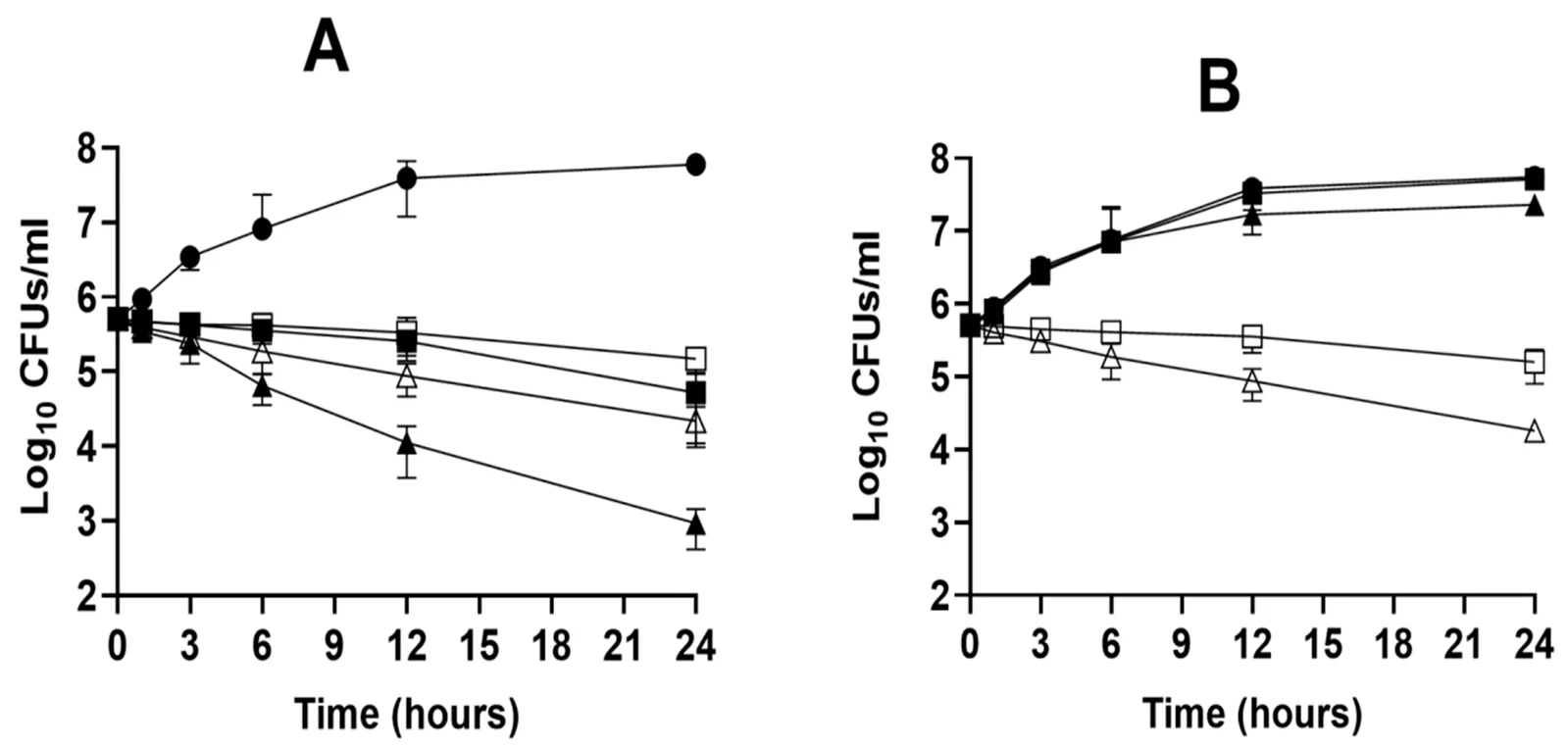
**Time–kill kinetics of WSEE against *A. baumannii*.** (**A**) Time–kill curves for the DS *A. baumannii* strain treated with WSEE (125 and 250 µg/mL) or colistin (1 and 2 µg/mL). Both WSEE and colistin produced a time-dependent reduction in bacterial viability over 24 h. (**B**) Time–kill curves for the CR *A. baumannii* isolate showing minimal response to colistin, while WSEE induced a marked, dose-dependent decrease in viable counts. Data are expressed as log_10_ CFU/mL over time. Control (⬤), Colistin-1 (⬛), Colistin-2 (▲), WSEE-125 (☐), and WSEE-250 (Δ).

The time–kill assay further substantiated the antibacterial efficacy of WSEE against both DS and CR *A. baumannii* strains by tracking changes in viable bacterial counts over a 24 h period. As shown in Figure 2A, exposure of the DS isolate to WSEE resulted in a progressive, time-dependent decline in colony-forming units (CFUs), comparable to the bactericidal profile observed with colistin. Treatment with colistin at 1 µg/mL led to a >3 log_10_ CFU/mL reduction, while 2 µg/mL achieved a >4 log_10_ CFU/mL decrease after 24 h.

In contrast, the CR *A. baumannii* isolate exhibited minimal susceptibility to colistin at both tested concentrations (1 and 2 µg/mL), as illustrated in Figure 2B. WSEE exhibited a concentration-dependent antibacterial effect. At 125 µg/mL, the extract produced a >2 log_10_ CFU/mL reduction in bacterial counts, indicating substantial antibacterial activity. Increasing the concentration to 250 µg/mL resulted in a >3 log_10_ CFU/mL reduction, consistent with bactericidal activity and supported by the corresponding MBC value.

### 2.5. WSEE Effectively Reduces Preformed Biofilms of A. baumannii

**Figure 3:**
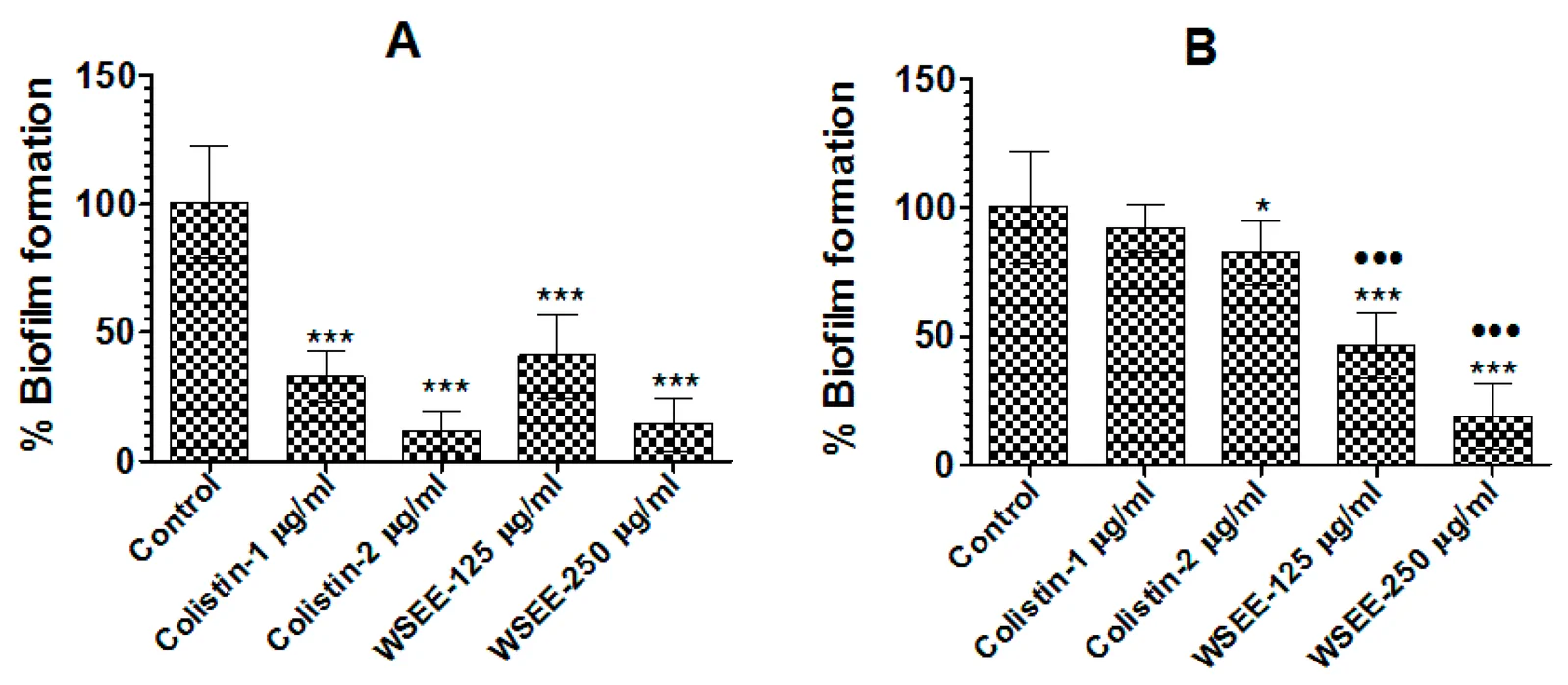
**WSEE Effectively Reduces Preformed Biofilms of *A. baumannii*.** (**A**) Percentage of biofilm formation by the DS *A. baumannii* strain following treatment with colistin (1 and 2 µg/mL) or WSEE (125 and 250 µg/mL). Treatments vs. the control group are indicated (*** *p* < 0.001). (**B**) Percentage biofilm formation by the CR *A. baumannii* isolate under the same treatment conditions. Data are expressed as mean ± SD. Treatments vs. control (* *p* < 0.05; *** *p* < 0.001), WSEE-125 or 250 µg/mL vs. colistin-2 µg/mL (●●● *p* < 0.001).

The antibiofilm efficacy of WSEE was evaluated in comparison to colistin against preformed biofilms of *A. baumannii* isolates. As shown in Figure 3A, colistin exhibited significant biofilm-disrupting activity against the DS strain, reducing biofilm biomass to 32.67% and 12% at concentrations of 1 µg/mL and 2 µg/mL, respectively (*p* < 0.001). However, as depicted in Figure 3B, colistin displayed minimal activity against preformed biofilms of CR *A. baumannii*, with no statistically significant reduction compared to the untreated control.

In contrast, WSEE demonstrated strong antibiofilm activity against both DS and CR *A. baumannii*. For the DS strain, WSEE at 125 µg/mL reduced biofilm formation to 41%, and at 250 µg/mL, biofilm biomass was diminished to 14% relative to the untreated control (Figure 3A). Remarkably, WSEE was also highly effective against preformed biofilms of the CR isolate, reducing biofilm mass to 46% at 125 µg/mL and 19% at 250 µg/mL (Figure 3B). These findings clearly indicate that WSEE possesses potent antibiofilm properties capable of disrupting established biofilms in both colistin-sensitive and CR *A. baumannii* strains, an effect not observed with colistin treatment. This underscores the potential of WSEE as a natural antibiofilm agent for controlling multidrug-resistant pathogens.

### 2.6. WSEE Promotes Leukocyte Recovery in Cyclophosphamide-Treated Mice

**Figure 4:**
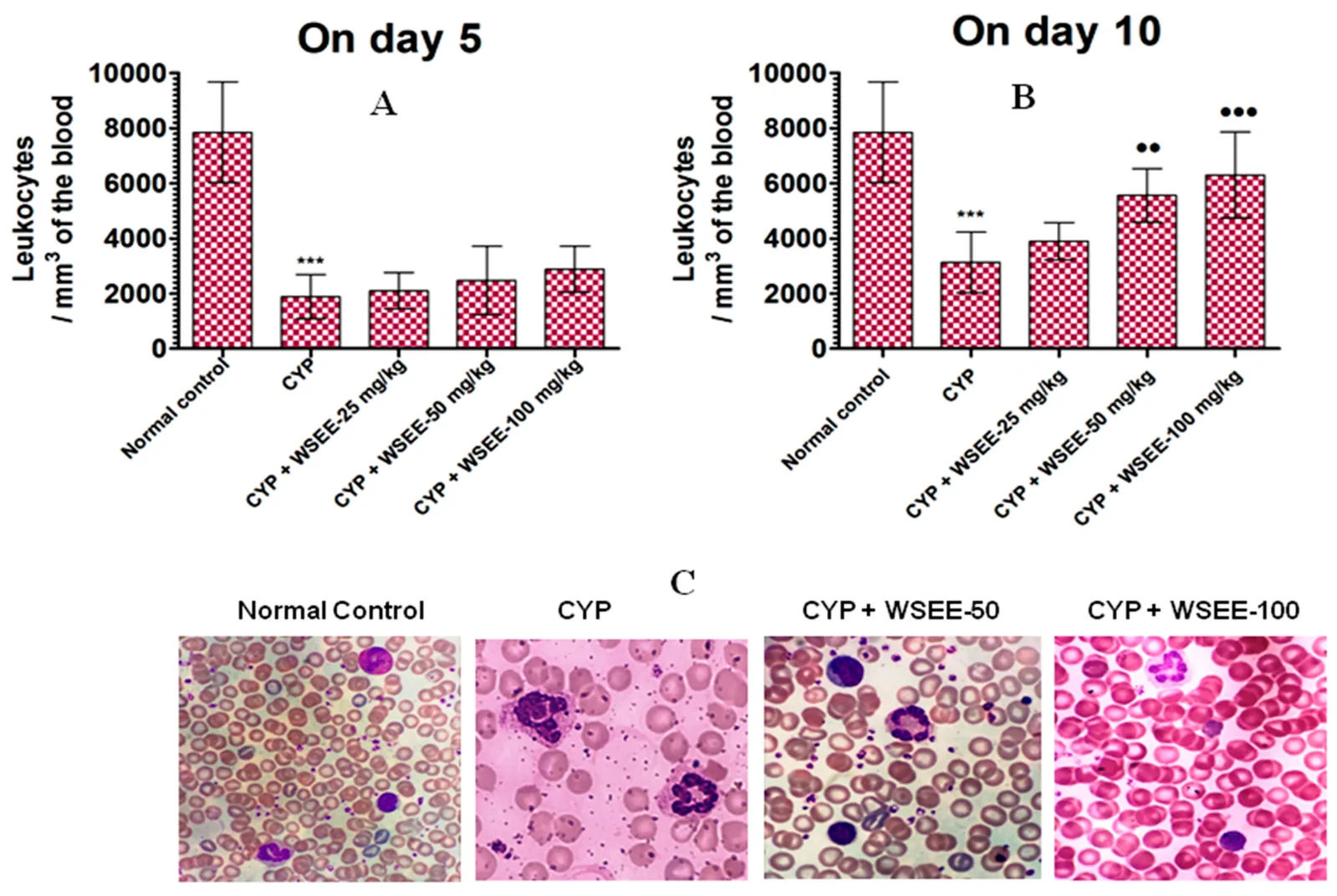
**WSEE restores leukocyte counts in CYP-induced leukopenic mice.** (**A**,**B**) Total leukocyte counts on days 5 and 10 after CYP treatment show significant leukopenia, which is dose-dependently reversed by WSEE. Data are presented as mean ± SD. *** *p* < 0.001 compared with the normal control group; ●● *p* < 0.01 and ●●● *p* < 0.001 compared with the CYP-treated group. (**C**) Representative Leishman-stained blood smears illustrating leukocyte depletion after CYP treatment and recovery of normal leukocyte morphology following WSEE administration. Data are mean ± SD; statistical significance is indicated in the figure.

The immune-restorative potential of WSEE was evaluated in a CYP-induced leukopenia model. As shown in Figure 4A,B, intraperitoneal administration of CYP (200 mg/kg) caused a marked reduction in total leukocyte count on days 5 and 10 post-treatment compared to normal controls, confirming successful induction of immunosuppression. Oral treatment with WSEE at 50 and 100 mg/kg markedly restored leukocyte counts in cyclophosphamide-treated mice, with the strongest effect observed on day 10. In contrast, the 25 mg/kg dose did not produce a statistically significant recovery. Treatment with WSEE (50 mg/kg) increased leukocyte counts from 3129 to 5560 cells/mm^3^ (*p* < 0.01), while WSEE (100 mg/kg) further elevated counts to 6301 cells/mm^3^ (*p* < 0.001) compared to the CYP group (Figure 4B).

Microscopic examination of blood smears supported these findings (Figure 4C). Leukocytes from normal control mice exhibited intact morphology with well-defined nuclei, whereas CYP-treated mice displayed severe cytoplasmic degranulation, nuclear fragmentation, and platelet aggregation, hallmarks of leukocyte damage. Notably, treatments with WSEE (50 and 100 mg/kg) showed marked structural restoration of leukocytes, indicating recovery of cellular integrity and immune function. These results collectively demonstrate that WSEE significantly counteracts CYP-induced leukopenia, enhancing both leukocyte count and morphology, thereby underscoring its immune-boosting potential.

### 2.7. Administration of WSEE Ameliorates CYP-Induced Hepato-Renal Toxicity in Mice

**Figure 5:**
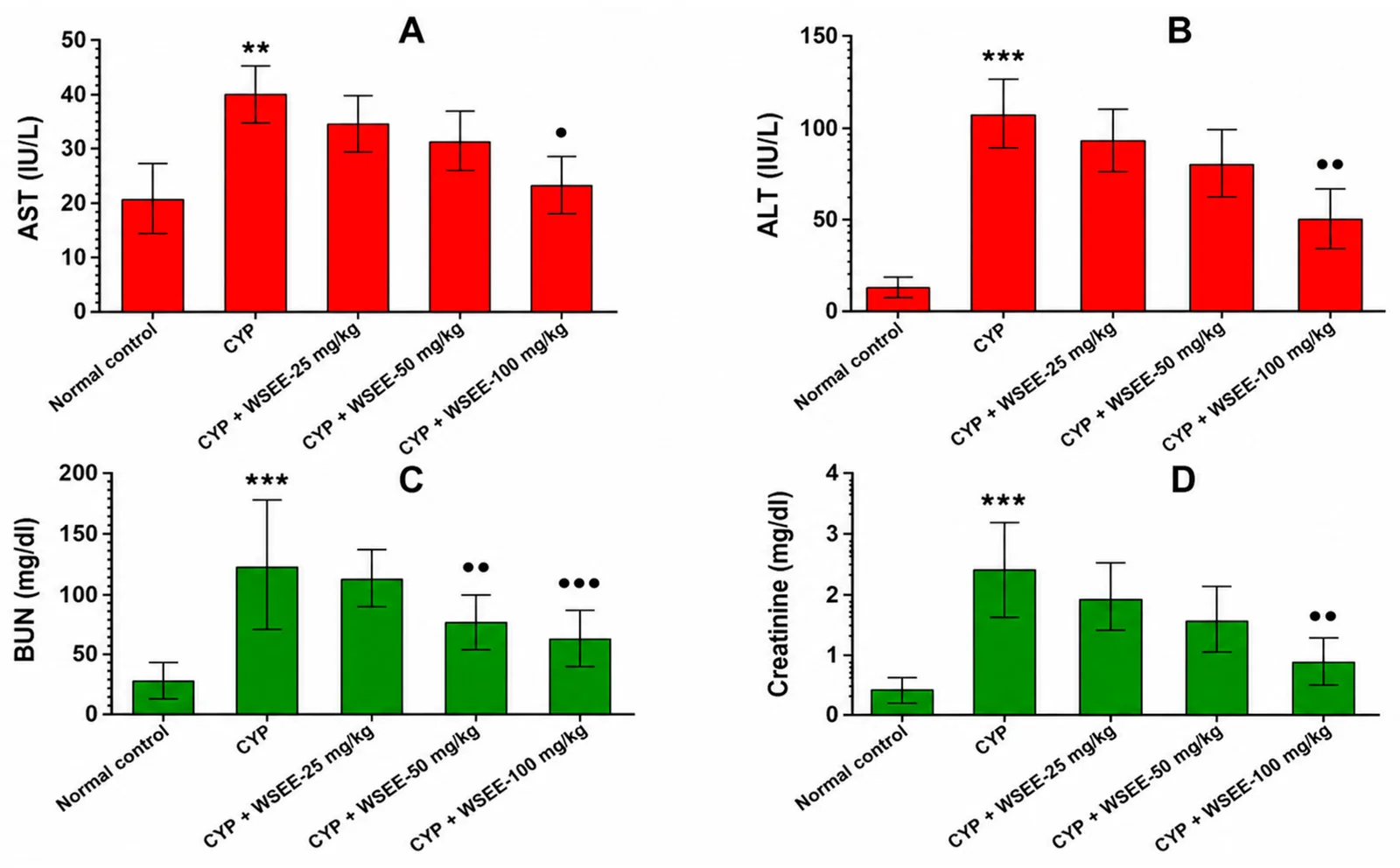
**Effect of WSEE on hepatic and renal function in CYP-treated mice.** Serum levels of (**A**) AST, (**B**) ALT, (**C**) BUN, and (**D**) creatinine were measured in normal control, CYP-treated, and CYP + WSEE. CYP induced significant hepatic and renal dysfunction, as evidenced by elevated biochemical markers, whereas WSEE treatment dose-dependently attenuated these alterations, indicating a protective effect. Data are presented as mean ± SD. ** *p* < 0.01 and *** *p* < 0.001 compared with the normal control group; ● *p* < 0.05, ●● *p* < 0.01 and ●●● *p* < 0.001 compared with the CYP-treated group.

CYP administration resulted in pronounced hepatic and renal toxicity, as evidenced by elevated serum levels of AST, ALT, BUN, and creatinine (Figure 5A–D). In normal mice, the AST level was 20 ± 7.2 IU/L, which significantly increased to 40.6 ± 5.7 IU/L in CYP-treated mice (*p* < 0.01). Treatment with WSEE at 100 mg/kg markedly reduced AST levels to 22.67 ± 5 IU/L (*p* < 0.05), indicating restoration of liver function (Figure 5A). Similarly, CYP administration caused a substantial elevation in ALT from 17 ± 3.5 IU/L in normal mice to 104.7 ± 17 IU/L (*p* < 0.001), confirming hepatic injury (Figure 5B). WSEE at lower doses (25 and 50 mg/kg) produced a partial, nonsignificant reduction in ALT levels, while treatment at 100 mg/kg significantly lowered ALT to 52.7 ± 7 IU/L (*p* < 0.01), demonstrating a strong hepatoprotective effect.

Renal function parameters also reflected the protective influence of WSEE. The BUN level increased from 26 ± 5.3 mg/dL in normal controls to 118 ± 17.8 mg/dL in CYP-injected mice (*p* < 0.001), indicating nephrotoxicity (Figure 5C). WSEE at 50 mg/kg reduced BUN to 77 ± 6 mg/dL (*p* < 0.01), and at 100 mg/kg, it further decreased to 60 ± 13 mg/dL (*p* < 0.001). Similarly, creatinine levels rose sharply from 0.53 ± 0.14 mg/dL in normal mice to 2.33 ± 0.61 mg/dL following CYP treatment (*p* < 0.001). Although WSEE at 25 and 50 mg/kg showed slight improvement, the changes were not statistically significant. Notably, WSEE at 100 mg/kg reduced creatinine to 0.90 ± 0.19 mg/dL (*p* < 0.01), confirming its nephroprotective action (Figure 5D). Taken together, these findings indicate that WSEE confers dose-dependent protection against CYP-induced hepatorenal damage. The extract effectively normalized serum biochemical markers, highlighting its potential as a natural hepatoprotective and nephroprotective agent.

### 2.8. WSEE Effectively Combated CR A. baumannii Infection in Leukopenic Mice

**Figure 6:**
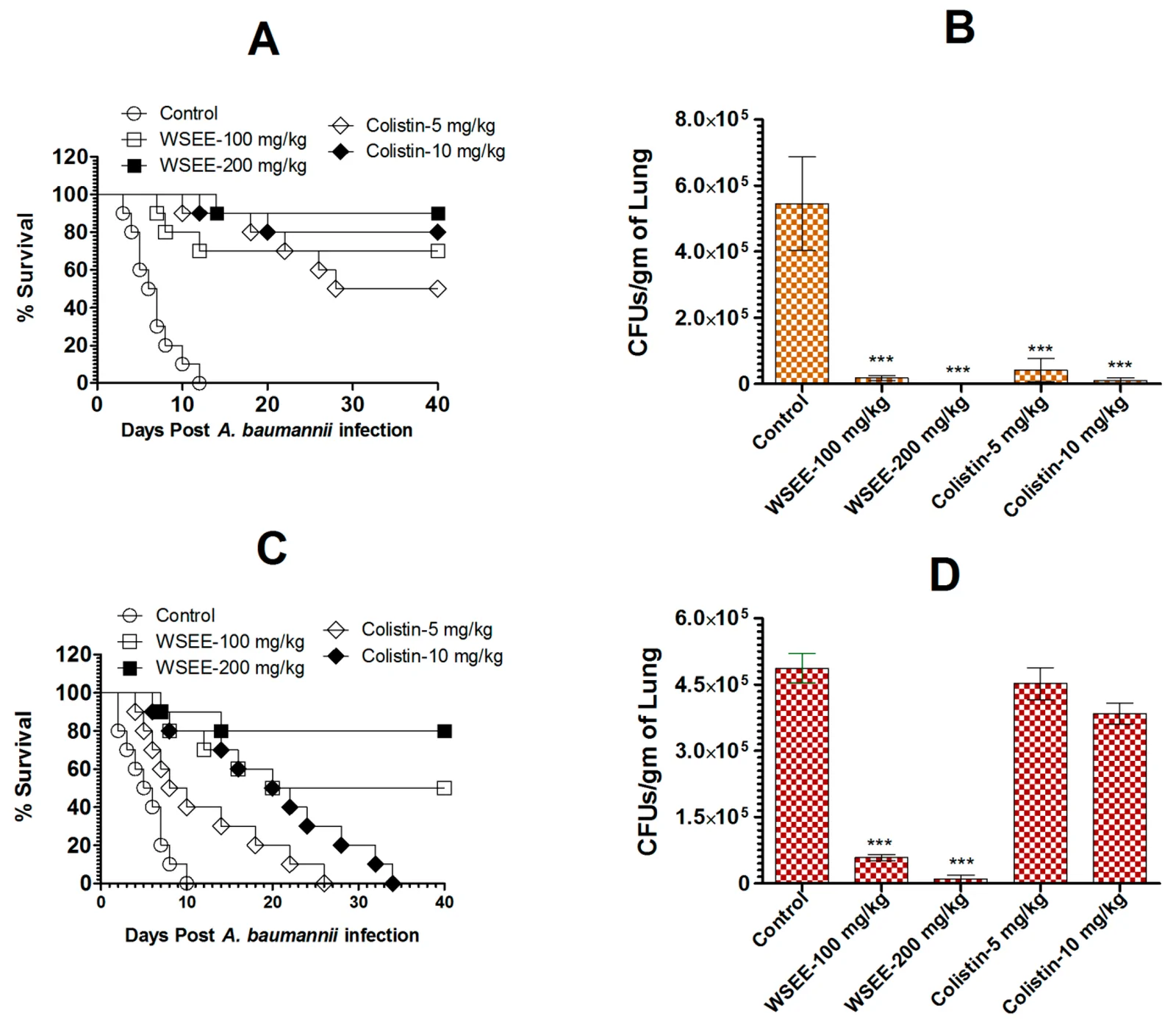
**Antibacterial efficacy of WSEE in leukopenic mice.** (**A**) Survival curve of mice infected with DS *A. baumannii* and treated with WSEE (100 and 200 mg/kg) or colistin (5 and 10 mg/kg). (**B**) Pulmonary bacterial burden (CFU/g lung) in mice infected with the DS strain on day 7 post-infection, showing significant reduction following WSEE or colistin treatment compared with untreated controls. (**C**) Survival curve of mice infected with CR *A. baumannii* and treated with WSEE or colistin. (**D**) Lung bacterial burden in mice infected with the CR strain showed substantial clearance with WSEE treatment, whereas colistin exhibited limited efficacy. Data are presented as mean ± SD. *** *p* < 0.001 compared with the control (untreated infected) group.

The efficacy of WSEE was evaluated in leukopenic mice challenged with either DS or CR *A. baumannii* and compared to the colistin treatment. In the leukopenic control group, *A. baumannii* infection proved fatal, with 100% mortality observed within 12 days (Figure 6A). Treatment with colistin at 5 mg/kg and 10 mg/kg significantly improved survival, yielding 50% and 80% survival rates, respectively (*p* < 0.0001). WSEE demonstrated a strong dose-dependent protective effect in this experimental model, achieving 60% survival at 100 mg/kg and 90% survival at 200 mg/kg (*p* < 0.0001).

Bacterial enumeration in lung tissues revealed a substantial reduction in bacterial burden among the treated groups (Figure 6B). Untreated infected mice exhibited 544,947 ± 57,152 CFU/lung, whereas treatment with colistin at 5 mg/kg and 10 mg/kg decreased CFUs to 41,621 ± 14,316 and 10,697 ± 3438, respectively (*p* < 0.0001). WSEE also markedly lowered pulmonary bacterial load, reducing it to 18,530 ± 2996 CFU at 100 mg/kg and to 1387 ± 640 CFU at 200 mg/kg (*p* < 0.0001), confirming its potent antibacterial action.

In animals infected with the CR *A. baumannii* strain, colistin treatment (5 or 10 mg/kg) failed to provide any protective effect, as all animals succumbed to infection within 40 days (Figure 6C). In contrast, WSEE significantly improved survival outcomes in a dose-dependent manner. WSEE at 100 mg/kg resulted in 50% survival by day 40 (*p* = 0.0002), while 200 mg/kg treatment increased survival to 70% (*p* < 0.0001). Consistent with the survival data, bacterial load analysis demonstrated that colistin had negligible impact on infection control, whereas WSEE significantly reduced lung bacterial counts (Figure 6D). WSEE at 100 mg/kg decreased the bacterial burden to 58,617 ± 12,110 CFU compared to 486,941 ± 57,202 CFU in untreated controls (*p* < 0.001). A higher dose of 200 mg/kg further reduced bacterial load to 10,764 ± 3514 CFU (*p* < 0.0001). Collectively, these findings establish that WSEE exhibits potent antibacterial efficacy against *A. baumannii* infections in immunocompromised hosts, significantly improving survival and reducing bacterial burden in a dose-dependent way.

### 2.9. Renal Safety Profile of WSEE and Colistin in A. baumannii-Infected Mice

**Figure 7:**
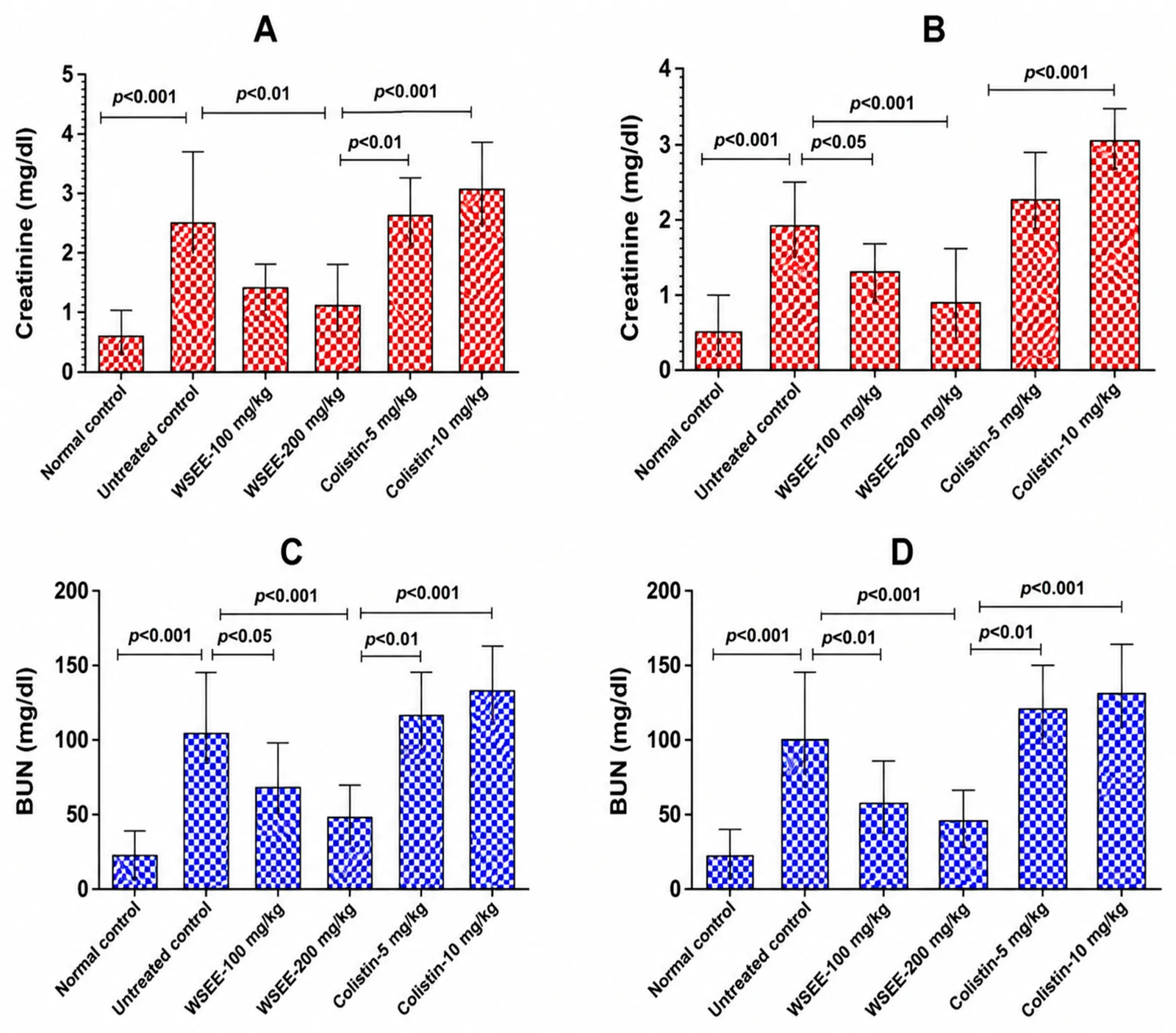
**Renal safety profile of WSEE and colistin in *A. baumannii*-infected leukopenic mice.** Serum creatinine (**A**,**B**) and BUN (**C**,**D**) levels were measured to assess renal function. Panels (**A**,**C**) represent figures for mice infected with DS *A. baumannii*, while panels (**B**,**D**) correspond to CR *A. baumannii*-infected mice. Untreated infected mice showed significant elevations in creatinine and BUN, indicating renal impairment. Treatment with WSEE (100 and 200 mg/kg) significantly attenuated these elevations, whereas colistin treatment was associated with higher renal toxicity markers. The results are presented as mean ± SD, with statistical significance indicated between groups.

The safety profiles of WSEE and colistin were evaluated by analyzing serum creatinine and BUN levels in infected DS or CR *A. baumannii* mice (Figure 7). Mice infected with DS *A. baumannii* exhibited markedly elevated serum creatinine from 0.52 ± 0.14 mg/dL in healthy controls to 2.3 ± 0.61 mg/dL (*p* < 0.001; Figure 7A). Treatment with WSEE significantly restored renal function, reducing creatinine levels to 1.3 ± 0.13 mg/dL at 100 mg/kg (*p* < 0.05) and 1.0 ± 0.28 mg/dL at 200 mg/kg (*p* < 0.01). In contrast, colistin administration worsened renal function, further increasing creatinine to 2.5 ± 0.21 mg/dL at 5 mg/kg and 2.9 ± 0.24 mg/dL at 10 mg/kg. A similar pattern was detected in CR *A. baumannii*-infected mice, in which colistin treatment further increased nephrotoxicity. Creatinine levels rose to 2.63 ± 0.35 mg/dL (5 mg/kg) and 3.2 ± 0.45 mg/dL (10 mg/kg; Figure 7B). Conversely, WSEE treatment effectively normalized renal parameters, with creatinine values of 1.3 ± 0.26 mg/dL (100 mg/kg) and 0.91 ± 0.27 mg/dL (200 mg/kg), showing no significant difference from normal mice.

BUN levels also reflected the nephroprotective advantage of WSEE (Figure 7C,D). Infected mice exhibited elevated BUN levels: 102 ± 15.7 mg/dL for DS and 101 ± 17.6 mg/dL for CR infections compared to 23.6 ± 4.9 mg/dL in normal controls (*p* < 0.001). Colistin administration further aggravated renal stress, elevating BUN to 131 ± 29 mg/dL in DS and 140 ± 35 mg/dL in resistant infections. In contrast, WSEE treatment produced a dose-dependent reduction in BUN levels. In DS infections, WSEE reduced BUN to 65 ± 13 mg/dL at 100 mg/kg and 47 ± 6.6 mg/dL at 200 mg/kg. Similarly, in CR infections, WSEE significantly decreased BUN from 101 ± 17.6 mg/dL to 58.6 ± 9.8 mg/dL at 100 mg/kg (*p* < 0.01) and to 45.6 ± 6.8 mg/dL at 200 mg/kg (*p* < 0.001). Collectively, these findings indicate that WSEE exhibited a favorable renal safety profile and renoprotective potential under the experimental conditions of this study. In contrast to colistin treatment under the present experimental conditions, WSEE preserved renal function and improved biochemical markers, supporting its biosafety and therapeutic advantage in *A. baumannii* infection models.

### 2.10. WSEE Reduces Serum Concentrations of IL-6 and TNF-α

**Figure 8:**
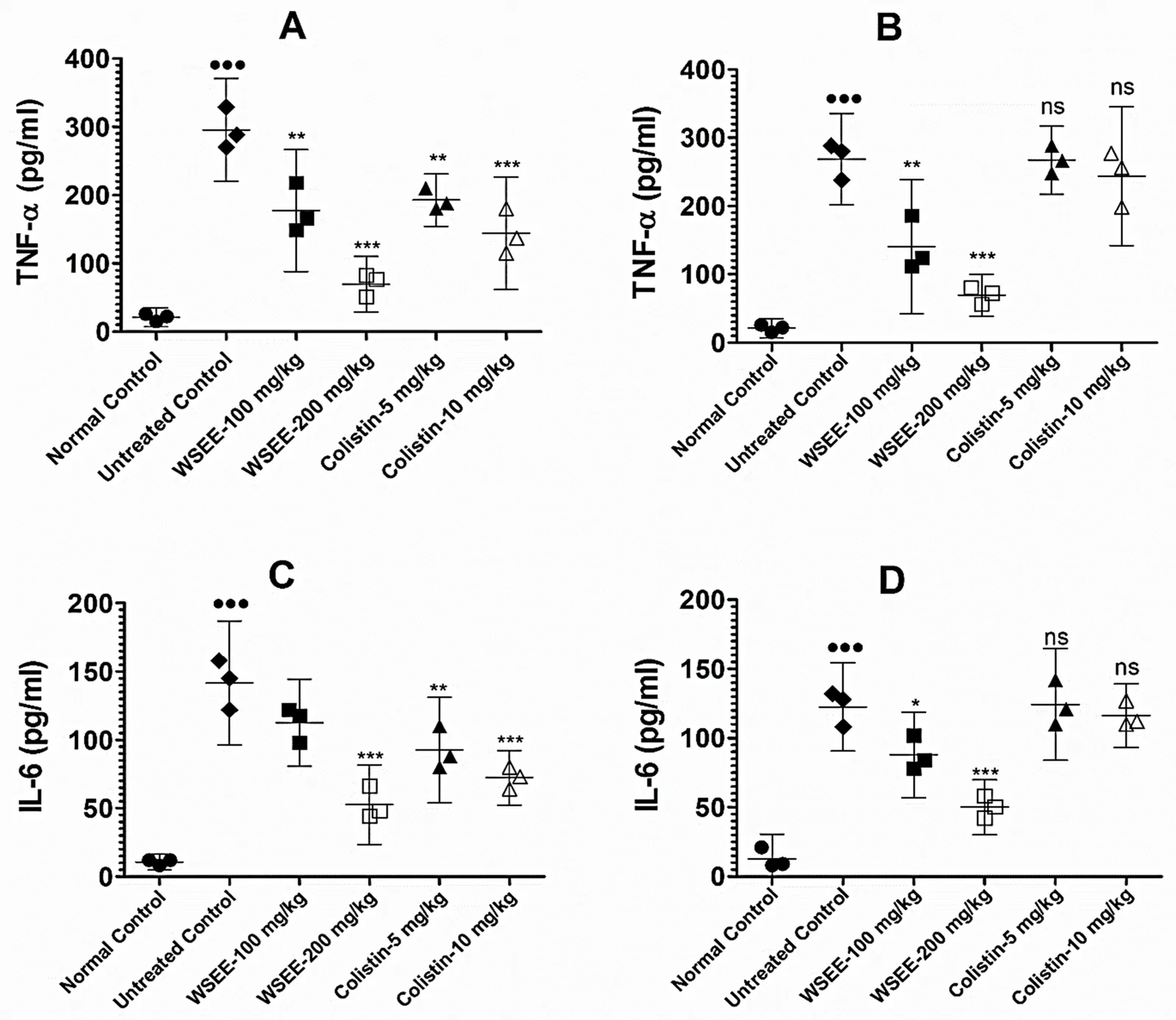
**Effect of WSEE on systemic inflammatory cytokines in *A. baumannii*-infected leukopenic mice.** TNF-α (**A**,**B**) and IL-6 (**C**,**D**) were quantified by ELISA in normal control, untreated infected, and treated groups. Panels (**A**,**C**) represent cytokine levels in DS *A. baumannii-*infected mice, whereas panels (**B**,**D**) show cytokine levels in mice infected with CR *A. baumannii*. Treatment with WSEE (100 and 200 mg/kg) significantly reduced pro-inflammatory cytokine levels compared to untreated controls and showed greater immunomodulatory efficacy than colistin. Data are expressed as mean ± SD. ●●● *p* < 0.001 (normal vs. untreated control); * *p* < 0.05, ** *p* < 0.01, and *** *p* < 0.001 (untreated control vs. treatment groups). “ns” signifies “Not Significant”.

The anti-inflammatory potential of WSEE was evaluated by measuring proinflammatory cytokines, such as TNF-α and IL-6, in mice infected with either DS or CR A. baumannii. Mice infected with the DS strain caused a substantial elevation of TNF-α (295 ± 30 pg/mL) compared to healthy controls (21 ± 5.5 pg/mL, *p* < 0.001; Figure 8A). WSEE ameliorated TNF-α levels in a dose-dependent way, reducing them to 177 ± 36 pg/mL at 100 mg/kg and 70 ± 16 pg/mL at 200 mg/kg (*p* < 0.01 and *p* < 0.001, respectively). Colistin also effectively reduced TNF-α levels, yielding 193 ± 15.6 pg/mL at 5 mg/kg and 144 ± 33 pg/mL at 10 mg/kg. However, in CR A. baumannii-infected mice, colistin treatment failed to significantly alter TNF-α levels (Figure 8B). In contrast, WSEE maintained its anti-inflammatory efficacy, lowering TNF-α from 269 ± 27 pg/mL in untreated mice to 141 ± 40 pg/mL (*p* < 0.01) at 100 mg/kg and to 69 ± 12 pg/mL (*p* < 0.001) at 200 mg/kg.

A similar pattern was observed for IL-6 (Figure 8C,D). In DS *A. baumannii*-infected mice, IL-6 levels increased to 142 ± 18 pg/mL versus 11 ± 2.3 pg/mL in healthy controls (*p* < 0.001). Colistin effectively reduced IL-6 to 93 ± 16 pg/mL at 5 mg/kg (*p* < 0.01) and 72 ± 8 pg/mL at 10 mg/kg (*p* < 0.001). Conversely, colistin had no significant impact on IL-6 production in mice infected with the CR strain. WSEE treatment, however, markedly suppressed IL-6 in these resistant infections, reducing it from 123 ± 13 pg/mL in untreated mice to 88 ± 12 pg/mL at 100 mg/kg and 50 ± 8 pg/mL at 200 mg/kg (*p* < 0.001). Overall, WSEE markedly reduced systemic TNF-α and IL-6 levels in *A. baumannii*-infected mice, including those infected with CR strains.

## 3. Discussion

*Acinetobacter baumannii* is responsible for a broad range of clinical infections in humans, such as pneumonia, bacteremia, urinary tract infections (UTIs), and wound-associated infections [3,45]. It has emerged as a major clinical challenge, particularly in intensive care units (ICUs), due to its remarkable antibiotic resistance and its ability to infect immunocompromised patients [2]. The ability of *A. baumannii* to persist on medical devices and environmental surfaces in healthcare settings promotes ongoing transmission and recurrent outbreaks. Therefore, controlling *A. baumannii* infections requires a multifaceted approach encompassing strict infection-control practices, continuous surveillance, and the development of novel therapeutic strategies [46].

The rapid evolution of antibiotic resistance in *A. baumannii* has critically reduced available treatment options. Conventional therapies often employ combinations of carbapenems, polymyxins (e.g., colistin), tigecycline, and sulbactam [47,48,49]. However, their efficacy depends largely on the susceptibility profile of the infecting strain. With the increasing prevalence of multidrug- and extensively drug-resistant isolates, alternative approaches, including bacteriophage therapy and monoclonal antibodies targeting *A. baumannii* surface antigens, are being actively investigated [50,51]. Likewise, novel agents including siderophore-conjugated antibiotics and antimicrobial peptides have shown encouraging results in preclinical investigations [52]. These developments underscore the urgent need for complementary and safer therapeutic options.

As a result, herbal medicines are receiving growing interest as potential alternatives or adjuncts to conventional antibiotic therapies. Several medicinal plants have demonstrated antimicrobial activity against *A. baumannii*, mainly attributed to the presence of flavonoids, alkaloids, terpenoids, and phenolic compounds [53]. Traditional botanicals, such as Japanese rose (*Rosa rugosa*), Myrobalan (*Terminalia chebula*), Baical skullcap (*Scutellaria baicalensis*), and Clove (*Syzygium aromaticum*), have shown notable inhibitory effects against *A. baumannii* [53]. In addition, plant-derived compounds, including thymoquinone, ellagic acid, baicalein, and eugenol, have exhibited potent antibacterial activities against drug-resistant *A. baumannii* [15,16].

The GC–MS analysis was performed to provide a preliminary qualitative phytochemical profile of the WSEE, thereby improving the chemical characterization and reproducibility of the extract used in this study. The biological activities listed in Supplementary Figure S1 are based on previously published reports and are included solely to provide phytochemical context rather than to attribute the observed antibacterial, antibiofilm, immunomodulatory, or therapeutic effects to individual constituents. However, because GC–MS primarily detects volatile and semi-volatile compounds, it does not comprehensively characterize thermolabile and non-volatile metabolites, including withanolides, which are among the principal bioactive constituents of *W. somnifera*. Likewise, the relatively high total phenolic and flavonoid contents of WSEE suggest that these phytochemicals may collectively contribute to its antibacterial and antioxidant activities. Nevertheless, TPC and TFC assays do not identify or quantify individual constituents. Therefore, further HPLC–MS/LC–MS-based phytochemical profiling combined with bioactivity-guided fractionation is required to identify the specific bioactive compounds and elucidate their relative contributions to the antibacterial, antibiofilm, immunomodulatory, and therapeutic effects observed in this study. Although WSEE demonstrated potent antibacterial activity against both DS and CR *A. baumannii,* the precise molecular targets responsible for these effects remain to be elucidated. Future studies should investigate whether WSEE disrupts bacterial cell membrane or cell wall integrity; interferes with protein or nucleic acid synthesis; and modulates the expression of antimicrobial resistance, quorum-sensing, and biofilm-associated genes using RT-qPCR, transcriptomic, and proteomic approaches.

The present study evaluated the antibacterial and immune-stimulatory activity of WSEE against both DS and CR *A. baumannii*. In vitro analyses, including agar well diffusion and MIC determination, demonstrated that WSEE exerted strong inhibitory effects against both isolates. The time–kill assay demonstrated a concentration-dependent antibacterial effect of WSEE. While 125 µg/mL produced a marked reduction in bacterial viability (>2 log_10_ CFU/mL), bactericidal activity (≥3 log_10_ CFU/mL reduction) was achieved at 250 µg/mL. Notably, WSEE significantly disrupted preformed biofilms, especially those formed by CR strains. Biofilm formation is a well-recognized survival strategy that enhances the resistance of *A. baumannii* to host immune defenses and antimicrobial therapy, thereby contributing to persistent infections and treatment failure. Therefore, disrupting established biofilms is an important therapeutic strategy for effective infection control. In the present study, WSEE significantly reduced the biomass of preformed biofilms, demonstrating its potential antibiofilm activity. Similar findings have been reported for *W. somnifera* against *Streptococcus mutans*, where its phytoconstituents inhibited bacterial adhesion and extracellular polysaccharide synthesis [54]. These observations suggest that WSEE may help overcome biofilm-associated antibiotic resistance. However, the crystal violet assay used in the present study measures total biofilm biomass and does not distinguish between biofilm disruption and killing of sessile bacteria. Therefore, future studies employing live/dead fluorescence staining, confocal laser scanning microscopy, and biofilm viability assays are warranted to better elucidate the mechanism underlying the antibiofilm activity of WSEE.

Immunocompromised individuals are particularly susceptible to *A. baumannii* infections because of reduced host immune defense, which can lead to persistent infection and treatment failure [55]. Restoration of immune function thus represents a critical strategy to combat *A. baumannii* [56,57]. In our study, CYP-induced leukopenic mice showed severe susceptibility to *A. baumannii* infection, whereas WSEE treatment restored leukocyte counts and ameliorated CYP-induced toxicity. The observed recovery in total leukocyte counts suggests that WSEE exerts immunomodulatory effects and promotes partial recovery of immune status in cyclophosphamide-induced leukopenic mice. The immunostimulatory potential of *W. somnifera* has been previously documented, and its methanolic extract elevates total white blood cell counts, enhances antibody titers, and activates macrophages [26]. Neutrophils are pivotal in defense against *A. baumannii*, producing reactive oxygen species (ROS), beta-defensins, and myeloperoxidase to mediate bacterial killing [58]. Thus, the leukocyte recovery induced by WSEE may have contributed to the improved bacterial clearance and survival observed in our study. Other investigations also support the immunomodulatory actions of *W. somnifera*, including increased Th1 cytokine and IgG2a production and protection against *Salmonella typhimurium* and *Listeria monocytogenes* [59,60,61]. Furthermore, synergistic effects of *W. somnifera* with antibiotics have been reported against *Escherichia coli* and *Salmonella typhi* [62], and its constituents have shown antifungal activity against *Fusarium oxysporum*, *Aspergillus flavus*, and *C. albicans* [63,64]. Although WSEE demonstrated favorable therapeutic effects in comparison to colistin in the present study, these findings should be interpreted with caution because the two treatments were administered via different routes. WSEE was given orally to reflect its intended use as a botanical therapeutic, whereas colistin was administered intraperitoneally according to established experimental protocols for systemic treatment of *A. baumannii* infection. Differences in absorption, bioavailability, and tissue distribution associated with these administration routes may have influenced the observed therapeutic outcomes. Therefore, the present results should not be interpreted as evidence of intrinsic superiority of WSEE over colistin. Future studies employing comparable administration routes together with pharmacokinetic and pharmacodynamic evaluations will be necessary to enable a more rigorous comparison of their therapeutic efficacy.

Inflammation is a major contributor to the pathogenesis of *A. baumannii* infection, particularly in pulmonary disease, where excessive production of pro-inflammatory cytokines, such as TNF-α, IL-6, and IL-1β, promotes tissue injury and disease progression [15]. Persistent IL-6 signaling further exacerbates chronic airway inflammation and tissue remodeling, as observed in chronic obstructive pulmonary disease (COPD) and other respiratory disorders, making it an attractive therapeutic target [65,66]. In the present study, WSEE significantly reduced serum levels of TNF-α and IL-6 in *A. baumannii*-infected mice, indicating its ability to attenuate the excessive inflammatory response associated with infection. These findings are consistent with previous clinical and experimental studies demonstrating that WSEE reduces IL-6 levels in patients with COPD [67] and suppresses TNF-α, IL-1β, and other inflammatory mediators in lipopolysaccharide-induced inflammatory models [68]. Although the present findings support the immunomodulatory potential of WSEE, future studies incorporating additional markers such as IL-10, myeloperoxidase (MPO) activity, neutrophil infiltration, and immune cell phenotyping will provide a more comprehensive understanding of the mechanisms underlying its protective effects.

Collectively, our findings indicate that WSEE exerts multiple beneficial effects against both DS and CR *A. baumannii*, including antibacterial, antibiofilm, anti-inflammatory, and immunomodulatory activities. These effects were associated with reduced bacterial burden and improved survival in infected leukopenic mice. In addition, biochemical analyses showed that WSEE did not produce detectable hepatotoxicity or nephrotoxicity, in contrast to colistin, which is well known for its dose-limiting nephrotoxicity [46]. Despite these encouraging findings, several limitations should be acknowledged. First, phytochemical characterization was primarily qualitative and based on GC–MS analysis. Second, immunomodulatory activity was assessed using total leukocyte counts and selected inflammatory cytokines (TNF-α and IL-6). Third, the molecular mechanisms underlying the antibacterial and immunomodulatory effects of WSEE remain to be elucidated. Finally, the findings are based on a murine model, and additional pharmacokinetic, toxicological, and clinical studies are required before translation to human use.

Overall, the present findings suggest that WSEE may represent a promising complementary therapeutic approach for drug-resistant *A. baumannii* infections. Future studies should focus on quantitative standardization of withanolides, elucidation of the underlying molecular mechanisms, evaluation of pharmacokinetic properties, and assessment of potential synergistic effects with conventional antibiotics to facilitate clinical translation.

## 4. Materials and Methods

Nutrient agar and tryptic soy broth were obtained from Condalab (Madrid, Spain), while all analytical-grade chemicals were purchased from Fisher Scientific (Hampton, NY, USA). Diagnostic kits for measuring aspartate transaminase (AST), alanine transaminase (ALT), creatinine, and blood urea nitrogen (BUN) were supplied by QCA (Amposta, Spain). *W. somnifera* (Ashwagandha) root powder (Product code: OGT-00391) was procured from iHerb (Uttar Pradesh, India) and utilized for the preparation of the extract used in subsequent experiments.

### 4.1. Preparation of W. somnifera Ethanolic Extract (WSEE)

Dried roots of *W. somnifera* were powdered and extracted using a modified Soxhlet extraction method [69]. Briefly, the powdered material was first defatted with cyclohexane under continuous stirring for 4 h to remove non-polar constituents. The defatted residue was then subjected to Soxhlet extraction with analytical-grade ethanol for 48 h (Buchi Laboratories, Flawil, Switzerland). Following extraction, the solvent was removed under reduced pressure using a rotary vacuum evaporator to obtain a semi-solid crude extract, which was subsequently air-dried at room temperature to a constant weight. The dried extract was weighed to determine the extraction yield, which was 10.8% (*w*/*w*) relative to the initial dry plant material, and the yield was used as a quality control parameter to ensure extraction reproducibility. Each extract batch was assigned a unique lot number and stored in airtight containers at −20 °C until further use to preserve its phytochemical integrity.

### 4.2. Determination of Flavonoid, Phenolic, and Antioxidant Capacity of WSEE

#### 4.2.1. Total Flavonoid Content (TFC)

The TFC of WSEE was determined using the aluminum chloride colorimetric method [69]. Quercetin solutions (0–100 μg/mL) were prepared in methanol to generate a standard calibration curve. Briefly, 0.5 mL of WSEE was mixed with 0.16 mL of 5% sodium nitrite, followed by the addition of 0.16 mL of 10% aluminum chloride. After incubation, 1 mL of 1 M sodium hydroxide was added, and the mixture was mixed thoroughly. Absorbance was measured at 510 nm using a UV–Vis spectrophotometer. The flavonoid content was calculated from the quercetin standard curve and expressed as milligrams of quercetin equivalents per gram of extract (mg QE/g WSEE).

#### 4.2.2. Total Phenolic Content (TPC)

The TPC of WSEE was estimated using the Folin–Ciocalteu method [69]. A calibration curve was prepared using gallic acid (1.4–1000 μg/mL). For the assay, 1.6 mL of WSEE or gallic acid standard was mixed with 0.2 mL of five-fold-diluted Folin–Ciocalteu reagent and 0.2 mL of 10% sodium carbonate solution. The reaction mixture was incubated for 30 min at room temperature in the dark. Absorbance was then recorded at 765 nm, and the total phenolic content was calculated from the gallic acid standard curve. The results were expressed as milligrams of gallic acid equivalents per gram of extract (mg GAE/g WSEE).

#### 4.2.3. DPPH Free Radical Scavenging Activity

The antioxidant activity of WSEE was evaluated using the DPPH free radical scavenging assay with slight modifications to a previously described method [69]. WSEE and the reference antioxidant, ascorbic acid, were dissolved in dimethyl sulfoxide (DMSO). In each well of a 96-well microplate, 20 μL of the test sample was mixed with 180 μL of a freshly prepared DPPH solution (100 μM). The reaction mixtures were incubated in the dark at room temperature for 30 min, after which the absorbance was measured at 517 nm using a microplate reader. The free radical scavenging activity was expressed as the IC_50_ value, defined as the concentration required to inhibit 50% of DPPH radicals. IC_50_ values were determined for WSEE (10–500 μg/mL) and ascorbic acid (1–20 μg/mL).

### 4.3. Gas Chromatography–Mass Spectrometry (GC–MS) Profiling of WSEE

The chemical constituents of WSEE were characterized using GC–MS following a previously reported method with minor modifications [69]. Analysis was performed using an Agilent 7890A gas chromatograph coupled to a 5975 mass selective detector and equipped with an HP-5MS capillary column (30 m × 0.25 mm i.d., 0.25 μm film thickness; Agilent Technologies, Santa Clara, CA, USA). Helium (99.999% purity) was used as the carrier gas at a constant flow rate of 1.0 mL/min. The injector temperature was maintained at 230 °C, and samples were injected in split mode (20:1). The oven temperature program was as follows: initial temperature of 40 °C held for 1 min, increased to 150 °C at 10 °C/minute, then to 280 °C at 5 °C/minute, and finally held at 280 °C for 8 min, giving a total run time of 40 min. The ion source temperature was maintained at 150 °C, and electron ionization (EI) was performed at 70 eV. Mass spectra were acquired over an *m*/*z* range of 50–550 with a solvent delay of 3 min. Compounds were identified by comparing the acquired mass spectra with those in the NIST 2009 mass spectral library.

### 4.4. Acinetobacter baumannii

A CR *A. baumannii* isolate (1501-20) and a DS strain were obtained from the Department of Microbiology, King Fahad Specialist Hospital, Buraydah, Saudi Arabia. Both isolates were routinely sub-cultured and maintained on nutrient agar (NA) plates under standard laboratory conditions.

### 4.5. Evaluation of the Activity of WSEE Against A. baumannii

The antibacterial potential of WSEE against *A. baumannii* was determined using a standard agar well diffusion assay, following an established protocol [70]. Nutrient agar plates were evenly seeded with the respective bacterial suspensions, and sterile wells measuring 8 mm in diameter were created in the agar. Aliquots (50 µL) of WSEE at doses of 200 µg and 400 µg were dispensed into the wells. Colistin sulfate was included as a reference antibiotic at concentrations of 20 µg for the DS isolate and 40 µg for the CR isolate, whereas 10% DMSO served as the solvent control. Following incubation at 37 °C for 24 h, antibacterial efficacy was quantified by measuring the inhibition zones surrounding each well.

The minimum inhibitory concentration (MIC) of WSEE was determined using a broth microdilution method adapted from the Clinical and Laboratory Standards Institute’s (CLSI) recommendations for antimicrobial susceptibility testing, with appropriate modifications for the evaluation of crude plant extracts [71]. Serial twofold dilutions of WSEE were prepared in nutrient broth, and 10% DMSO was included as the solvent control. The MIC was defined as the lowest concentration showing complete inhibition of visible bacterial growth after 24 h of incubation at 37 °C. A series of twofold dilutions of WSEE, ranging from 2 to 2000 µg/mL, was prepared in nutrient broth. Actively growing *A. baumannii* cultures were standardized to a 0.5 McFarland turbidity (approximately 1 × 10^8^ CFUs/mL) and inoculated into broth tubes containing increasing concentrations of the extract. The cultures were incubated at 37 °C for 24 h, and the MIC was defined as the lowest concentration of WSEE that completely prevented visible bacterial growth. For comparison, the MIC of colistin against *A. baumannii* was determined using the automated VITEK^®^ system (bioMérieux, Lyon, France).

To determine the minimum bactericidal concentration (MBC), aliquots from wells showing no visible bacterial growth in the MIC assay were sub-cultured onto Mueller–Hinton agar plates and incubated at 37 °C for 24 h. The lowest concentration showing no bacterial colony growth was recorded as the MBC.

### 4.6. Time–Kill Kinetics Assay

The bactericidal activity of WSEE against *A. baumannii* was evaluated using a time–kill kinetics assay [70]. Exponentially growing bacterial cultures, adjusted to approximately 5 × 10^5^ CFUs/mL, were treated with WSEE at final concentrations of 125 µg/mL and 250 µg/mL. For comparison, parallel cultures were exposed to colistin at concentrations of 1 µg/mL and 2 µg/mL. All treatments were carried out in nutrient broth, and cultures were maintained at 37 °C under continuous agitation. At specified intervals (0, 3, 6, 12, and 24 h), duplicate samples (0.5 mL) were withdrawn, pelleted by centrifugation at 10,000 rpm for 15 min, and washed with sterile phosphate-buffered saline (PBS) to eliminate residual antimicrobial agents. The washed suspensions were serially diluted and spread onto nutrient agar plates, followed by incubation at 37 °C for 24 h. Bacterial survival was quantified by counting colony-forming units (CFUs), with a lower limit of detection of 100 CFUs/mL.

### 4.7. Effect of WSEE on Preformed Biofilms of A. baumannii

The ability of WSEE to disrupt preformed biofilms of *A. baumannii* was evaluated using a standard crystal violet microtiter plate assay, as previously described [70]. Briefly, bacterial cultures were propagated in nutrient broth to reach a density of approximately 1 × 10^6^ CFU/mL. Portions (100 µL) of the bacterial suspension were transferred into sterile 96-well polystyrene microplates and incubated at 37 °C for 24 h to permit biofilm development.

After the initial biofilm was established, the supernatant was carefully removed and replaced with fresh nutrient broth supplemented with WSEE (125 or 250 µg/mL) or colistin (1 or 2 µg/mL), ensuring minimal disturbance to the adherent biofilm layer. The plates were then incubated for an additional 24 h at 37 °C. Subsequently, non-adherent cells were gently removed by washing the wells with PBS, and the plates were air-dried for 20 min. The remaining biofilm biomass was stained with 0.1% crystal violet for 15 min, followed by thorough rinsing with PBS to eliminate excess dye. The bound stain was dissolved in 95% ethanol, and biofilm formation was quantified by measuring absorbance at 595 nm using a BIO-TEK EL310 microplate reader (BioTek Instruments, Winooski, VT, USA).

### 4.8. Experimental Animals

Male Swiss albino mice (6–8 weeks old, weighing 22–25 g) were acclimatized for one week before the start of the experiments and then randomly assigned to the experimental groups (*n* = 10 per group). Mice were housed under specific pathogen-free conditions in a controlled environment (22 ± 2 °C) with a 12 h light/12 h dark cycle and were provided with standard laboratory chow and water ad libitum. All animal experiments were conducted in accordance with institutional and internationally accepted guidelines for the care and use of laboratory animals and are reported in compliance with the ARRIVE 2.0 guidelines. The study protocol was approved by the Research Ethics Committee of the Deanship of Scientific Research, Qassim University. Animals were monitored at least twice daily for clinical signs of illness. Predefined humane endpoints included severe lethargy, inability to access food or water, loss of more than 20% of initial body weight, or severe respiratory distress. Animals meeting any of these criteria were humanely euthanized to minimize pain and suffering.

### 4.9. Evaluation of WSEE in Cyclophosphamide (CYP)-Induced Immunosuppression and Toxicity

A leukopenic mouse model was established by intraperitoneal administration of CYP at a dose of 200 mg/kg, as described previously [70]. Twenty-four hours after CYP administration, mice (*n* = 10 in each group) received oral doses of WSEE at 25, 50, or 100 mg/kg once daily for seven consecutive days. Blood samples were collected on days 5 and 10 following CYP treatment to quantify total leukocyte counts using an automated hematology analyzer. Additionally, peripheral blood smears were prepared from animals in the CYP-only and CYP + WSEE groups, air-dried, and stained with Leishman stain for 5 min. The stained smears were examined under a light microscope to assess leukocyte morphology, including nuclear structure, cytoplasmic granulation, and cellular integrity, in order to evaluate the impact of WSEE on cyclophosphamide-induced leukopenia.

To assess the impact of WSEE on hepatic and renal function in cyclophosphamide-treated mice, blood samples were collected from animals at the end of the treatment period. Serum was separated and analyzed for AST, ALT, BUN, and creatinine levels. The biochemical parameters were compared among normal control, CYP-treated, and CYP + WSEE groups to assess CYP-induced organ dysfunction and the potential protective effect of WSEE. WSEE was initially screened at doses of 25, 50, and 100 mg/kg for immune-stimulating activity in cyclophosphamide-induced leukopenic mice. Based on the dose–response results, 100 and 200 mg/kg were selected for subsequent therapeutic efficacy studies in *A. baumannii*-infected mice.

### 4.10. Mouse Model of Acinetobacter baumannii Infection and Therapeutic Evaluation of WSEE

Following acclimatization, mice were randomly assigned to the experimental groups using a random allocation method. A leukopenic infection model was established, as described above. Two independent in vivo infection models were then constructed by intravenously challenging separate groups of leukopenic mice with either the DS or the CR *A. baumannii* isolate at a dose of 1 × 10^7^ CFU per mouse, following a previously established protocol [15]. Twenty-four hours after infection, therapeutic interventions were initiated to evaluate the efficacy of WSEE and colistin independently in each infection model.

The therapeutic efficacy of WSEE was evaluated at oral doses of 100 and 200 mg/kg, whereas colistin was administered intraperitoneally at doses of 5 and 10 mg/kg. WSEE was freshly suspended in 0.5% carboxymethyl cellulose (CMC) immediately before administration, and control animals received an equivalent volume of the vehicle alone. For each bacterial strain (DS and CR *A. baumannii*), mice (*n* = 10 per group) were randomly allocated into five groups: (1) untreated infected control, (2) WSEE (100 mg/kg), (3) WSEE (200 mg/kg), (4) colistin (5 mg/kg), and (5) colistin (10 mg/kg). Although treatment administration could not be blinded because of the nature of the interventions, laboratory analyses and data interpretation were performed in an objective and standardized manner to minimize potential bias.

Therapeutic efficacy was assessed by monitoring animal survival daily for 30 days. To determine bacterial burden, three mice from each group were euthanized on day 7 after treatment initiation. The lungs were aseptically excised, weighed, and homogenized in sterile phosphate-buffered saline (PBS). Serial dilutions of the homogenates were plated onto nutrient agar and incubated at 37 °C for 24 h. The CFUs were enumerated, and pulmonary bacterial burden was calculated by multiplying the colony count by the corresponding dilution factor, as previously described [15].

### 4.11. Evaluation of Treatment Safety

Blood samples were collected from three mice in each treatment group on day 7 after treatment to assess hepatic and renal function. Serum concentrations of AST, ALT, creatinine, and BUN were measured using commercially available diagnostic kits according to the manufacturers’ instructions [16]. These parameters were evaluated to identify any potential hepatotoxic or nephrotoxic effects associated with WSEE or colistin treatment.

### 4.12. Status of Inflammation Markers in Mouse Serum

The impact of WSEE and colistin on systemic inflammation was assessed by determining serum concentrations of TNF-α and IL-6. Cytokine levels were quantified using commercially available ELISA kits (Abcam Inc., Cambridge, UK) according to the manufacturer’s recommended protocols.

### 4.13. Statistical Analyzes

Survival outcomes were evaluated using the Kaplan–Meier method, and statistical differences between survival curves were assessed using the log-rank (Mantel–Cox) test. Quantitative data, including bacterial burden and other experimental parameters, are presented as mean ± standard deviation (SD) and were analyzed using one-way analysis of variance (ANOVA), followed by Tukey’s multiple comparisons post hoc test. Statistical analyses were performed using GraphPad Prism software (version 6.0; GraphPad Software, La Jolla, CA, USA). A *p*-value of <0.05 was considered statistically significant.

## 5. Conclusions

This study demonstrates the potential therapeutic value of WSEE against both DS and CR *A. baumannii* infections. WSEE exhibited significant antibacterial and antibiofilm activities in vitro, reducing bacterial burden and disrupting preformed biofilms associated with persistent infections and reduced antibiotic susceptibility. In vivo, WSEE improved the survival of infected leukopenic mice and reduced bacterial burden, suggesting a protective effect in this experimental model. WSEE also exhibited immunomodulatory activity, as evidenced by enhanced leukocyte recovery and decreased serum levels of TNF-α and IL-6, indicating its ability to modulate the host inflammatory response during *A. baumannii* infection. Collectively, these findings suggest that WSEE may represent a promising complementary therapeutic strategy for multidrug-resistant *A. baumannii* infections. However, further studies are required to identify the active phytoconstituents; elucidate the underlying molecular mechanisms; and evaluate the pharmacokinetics, safety, and clinical efficacy of WSEE before its therapeutic potential can be fully established.

## Figures and Tables

**Table 1:** Major bioactive compounds in WSEE and their antimicrobial therapeutic effects.

Compound Name	Mol. wt.	Pharmacological Activity	Reference
Glutinol	427	Antimicrobial activity	[31]
Tetrapentacontane	914	Antimicrobial activity	[32]
9,19-Cyclolanostan-3-ol, aceate, (3*β*)-	470	Antifungal activity	[33]
Hexatriacontane	507	Antimicrobial activity	[34]
1-Docosanol	326	Antiviral activity	[35]
Silanamine, N-[(4-ethoxyphenyl)methyl]-1,1,1-trimethyl-	209	Antibacterial activity	[36]
2,4-Di-tert-butylphenol	206	Antibacterial activity	[37]
Octadecanoic acid	284	Antibacterial activity	[38]
Quinoline	129	Antimalarial activity	[39]
1,4-Dibromo-2,3-butanediol	246	Antiprotozoan activity	[40]
4*H*-Pyran-4-one	96	Antibacterial	[41]
Pentadecane	212	Antiparasitic activity	[42]
β-Sitosterol	415	Antimicrobial activity	[43]
Lupeol	427	Antiparasitic activity	[44]

## Data Availability

The original contributions presented in this study are included in the article.

## Supplementary Materials

The following supporting information can be downloaded at: https://www.mdpi.com/article/10.3390/ijms27167321/s1, Figure S1: Gas chromatography–mass spectrometry (GC–MS) chromatogram and phytochemical profile of WSEE. The upper panel shows the representative GC–MS chromatogram with eleven major peaks corresponding to the predominant volatile phytoconstituents identified in the extract. The lower panel summarizes the retention time, identified compounds, molecular formula, molecular weight, chemical class, and reported biological activities based on published literature.
