# Supplementary material for: Antibacterial and Immunomodulatory Effects of Withania somnifera Ethanolic Root Extract Against Colistin-Resistant Acinetobacter baumannii in Leukopenic Mice
Source: Int J Mol Sci. 2026 Aug 16;27(16):7321. doi: 10.3390/ijms27167321 (PMC13513214; doi:10.3390/ijms27167321)
Supplement: Supplementary file 1 [file ijms-27-07321-s001.zip › ijms-4469689-supplementary.pdf]

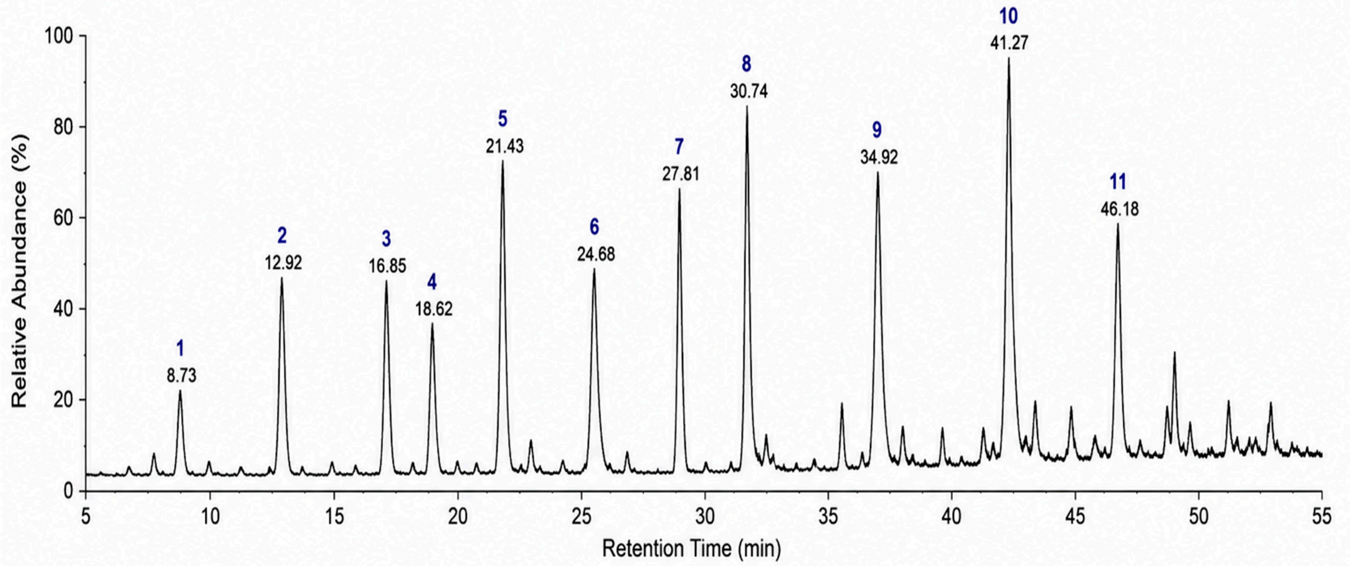

Peak No.	Retention Time (min)	Compound Identified	Molecular Formula	Molecular Weight (g/mol)	Nature / Class	Reported Activity
1	8.73	2-Furanmethanol, tetrahydro-5-methyl-	$C_6H_{12}O_2$	116.16	Furan derivative	Antioxidant
2	12.92	5-Hydroxymethylfurfural (HMF)	$C_6H_6O_3$	126.11	Furan derivative	Antimicrobial, antioxidant
3	16.85	Diethyl phthalate	$C_{12}H_{14}O_4$	222.24	Phthalate ester	Antimicrobial
4	18.62	1,2-Benzenedicarboxylic acid, diethyl ester	$C_{12}H_{14}O_4$	222.24	Aromatic ester	Antimicrobial
5	21.43	Hexadecanoic acid (Palmitic acid)	$C_{16}H_{32}O_2$	256.42	Saturated fatty acid	Antimicrobial, anti-inflammatory
6	24.68	9,12-Octadecadienoic acid (Linoleic acid)	$C_{18}H_{32}O_2$	280.45	Polyunsaturated fatty acid	Antioxidant, anti-inflammatory
7	27.81	Oleic acid	$C_{18}H_{34}O_2$	282.47	Monounsaturated fatty acid	Antimicrobial, anti-inflammatory
8	30.74	Octadecanoic acid (Stearic acid)	$C_{18}H_{36}O_2$	284.48	Saturated fatty acid	Anti-inflammatory
9	34.92	Squalene	$C_{30}H_{50}$	410.72	Triterpene	Antioxidant, anticancer
10	41.27	Phytol	$C_{20}H_{40}O$	296.53	Diterpene alcohol	Antimicrobial, anti-inflammatory
11	46.18	Stigmasta-5,22-dien-3-ol (β-Sitosterol)	$C_{29}H_{50}O$	414.71	Phytosterol	Anti-inflammatory, antioxidant

**Compounds identified based on GC–MS library (NIST) matching. Retention times may vary slightly depending on GC–MS conditions.*

Supplementary Figure S1. Gas chromatography–mass spectrometry (GC–MS) chromatogram and phytochemical profile of WSEE. The upper panel shows the representative GC–MS chromatogram with eleven major peaks corresponding to the predominant volatile phytoconstituents identified in the extract. The lower panel summarizes the retention time, identified compounds, molecular formula, molecular weight, chemical class, and reported biological activities based on published literature.
